# Prediction Model for Tumor Budding Status Using the Radiomic Features of F-18 Fluorodeoxyglucose Positron Emission Tomography/Computed Tomography in Cervical Cancer

**DOI:** 10.3390/diagnostics11081517

**Published:** 2021-08-23

**Authors:** Gun Oh Chong, Shin-Hyung Park, Shin Young Jeong, Su Jeong Kim, Nora Jee-Young Park, Yoon Hee Lee, Sang-Woo Lee, Dae Gy Hong, Ji Young Park, Hyung Soo Han

**Affiliations:** 1 Department of Obstetrics and Gynecology, School of Medicine, Kyungpook National University, Daegu 41944, Korea; gochong@knu.ac.kr (G.O.C.); sujeong1129@naver.com (S.J.K.); mylyh@naver.com (Y.H.L.); chssa0220@hanmail.net (D.G.H.); 2 Department of Obstetrics and Gynecology, Chilgok Hospital, Kyungpook National University, Daegu 41404, Korea; 3 Clinical Omics Research Center, School of Medicine, Kyungpook National University, Daegu 41944, Korea; pathpjy@naver.com (N.J.-Y.P.); hshan@knu.ac.kr (H.S.H.); 4 Department of Radiation Oncology, School of Medicine, Kyungpook National University, Daegu 41944, Korea; shinhyungpark@knu.ac.kr; 5 Cardiovascular Research Institute, School of Medicine, Kyungpook National University, Daegu 41944, Korea; 6 Department of Nuclear Medicine, School of Medicine, Kyungpook National University, Daegu 41944, Korea; swleenm@knu.ac.kr; 7 Department of Nuclear Medicine, Chilgok Hospital, Kyungpook National University Daegu, Daegu 41404, Korea; 8 Department of Pathology, School of Medicine, Kyungpook National University, Daegu 41944, Korea; jyparkmd@knu.ac.kr; 9 Department of Physiology, School of Medicine, Kyungpook National University, Daegu 41944, Korea

**Keywords:** cervical cancer, tumor budding, radiomic features, ^18^F-FDG PET/CT, prediction model

## Abstract

Objective: To compare the radiomic features of F-18 fluorodeoxyglucose positron emission tomography/computed tomography (^18^F-FDG PET/CT) and intratumoral heterogeneity according to tumor budding (TB) status and to develop a prediction model for the TB status using the radiomic feature of ^18^F-FDG PET/CT in patients with cervical cancer. Materials and Methods: Seventy-six patients with cervical cancer who underwent radical hysterectomy and preoperative ^18^F-FDG PET/CT were included. We assessed the status of intratumoral budding (ITP) and peritumoral budding (PTB) in all available hematoxylin and eosin-stained specimens. Three conventional metabolic parameters and fifty-nine features were extracted and analyzed. Univariate analysis was used to identify significant metabolic parameters and radiomic findings for TB status. The prediction model for TB status was built using 3 machine learning classifiers (random forest, support vector machine, and neural network). Results: Univariate analysis led to the identification of 2 significant metabolic parameters and 12 significant radiomic features according to intratumoral budding (ITB) status. Among these parameters, following multivariate analysis for the ITB status, only compacity remained significant (odds ratio, 5.0047; 95% confidence interval, 1.1636–21.5253; *p* = 0.0305). Two conventional metabolic parameters and 25 radiomic features were selected by the Lasso regularization, and the prediction model for the ITB status had a mean area under the curve of 0.762 in the test dataset. Conclusion: Radiomic features of ^18^F-FDG PET/CT were associated with the ITB status. The prediction model using radiomic features successfully predicted the TB status in patients with cervical cancer. The prediction models for the ITB status may contribute to personalized medicine in the management of patients with cervical cancer.

## 1. Introduction

Tumor budding (TB) is defined as a single neoplastic cell or cell cluster of up to four neoplastic cells at the invasive front of the tumor (peritumoral budding (PTB)) or within the tumor mass (intratumoral budding (ITB)) [1]. Several studies have demonstrated that TB is associated with lymphovascular invasion (LVI), lymph node metastasis, disease recurrence, and an unfavorable survival outcome, especially in colorectal cancer [2], esophageal carcinoma [3], and head and neck cancer [4]. Recently, we evaluated the prognostic roles of TB and the correlation between TB and conventional pathological parameters in gynecological cancers [5,6]. Our results demonstrated that TB was associated with deep depth invasion, higher International Federation of Gynecologic Obstetrics (FIGO) stage, LVI, and lymph node metastasis in endometrial cancer [5]. Moreover, high TB was an independent prognostic factor for predicting survival outcomes in cervical cancer [6].

Currently, F-18 fluorodeoxyglucose positron emission tomography/computed tomography (^18^F-FDG PET/CT) is widely used to detect lymph node involvement, distant metastasis, and recurrence in cervical cancer [7]. Various metabolic parameters of ^18^F-FDG PET/CT have been reported as prognostic factors, including the maximum standardized uptake value (SUVmax), metabolic tumor volume (MTV), and total lesion glycolysis (TLG). A recent systematic review showed that a high primary tumor SUVmax has a significant correlation with the poor event-free survival (hazard ratio (HR), 1.938; 95% confidence interval (CI), 1.203–3.054, *p* = 0.004) and overall survival (HR, 2.582; 95% CI, 1.936–3.443, *p* < 0.001). Moreover, the highest primary tumor TLG (HR, 1.843; 95% CI, 1.100–3.086, *p* = 0.02) and MTV (HR, 2.06; 95% CI, 1.21–3.51, *p* = 0.007) was associated with poor event-free survival [8]. Recently, radiomics studies, which represent intratumoral heterogeneity, have emerged as a new and exciting area of research. The measurement of texture indices from tumor ^18^F-FDG PET/CT images has recently been proposed as an adjunct to predict tumor response to therapy. Moreover, there is emerging evidence that intratumoral metabolic heterogeneity on pretreatment ^18^F-FDG PET/CT might be a predictor of tumor recurrence after treatment in patients with lung, esophageal, head and neck, and cervical cancer [9,10,11,12]. Furthermore, recent studies have shown that ^18^F-FDG PET/CT radiomics using various textural features are potential biomarkers to predict tumor recurrence and lymph node metastasis [13,14].

Tumor heterogeneity is defined as the presence of different cell subpopulations or clones and has a fundamental role in tumor growth, progression, and therapeutic resistance. Tumor hypoxia, angiogenesis, necrosis, fibrosis, cell proliferation, and inflammation are all known to affect tumor heterogeneity [15]. Epithelial–mesenchymal transition (EMT) of primary tumor tissues may lead to the loss of cell-to-cell adhesion, allowing individual cells or small groups of cells to acquire the ability to migrate and invade through the surrounding tissues. Moreover, TB may lead to more aggressive clinicopathologic characteristics through a similar mechanism of EMT, such as increased extracellular matrix degradation, increased migration, and loss of cell adhesion [16]. These EMT processes are accompanied by changes in cell morphology [17] and may lead to changes in tumor heterogeneity. However, to our knowledge, no previous studies have evaluated the correlation between metabolic parameters and radiomic findings of ^18^F- FDG PET/CT and TB in cervical cancer. Therefore, we hypothesized that TB may be associated with higher metabolic parameters in ^18^F-FDG PET/CT because of the aggressive behavior of TB and that the radiomic finding may differ according to TB status.

This study compared the radiomic features on ^18^F-FDG PET/CT and intratumoral heterogeneity according to TB status to develop a prediction model for the TB status using radiomic features of ^18^F-FDG PET/CT in patients with cervical cancer.

## 2. Materials and Methods

### 2.1. Patients

Following approval from the Institutional Review Board of Kyungpook National University Chilgok Hospital (KNUCH 2020-03-011), we reviewed the archival medical records and hematoxylin and eosin (H&E)-stained slides of patients with early-stage and locally advanced cervical cancer. The need for informed consent was waived due to the retrospective nature of the study. Between March 2011 and July 2015, a total of 136 patients who underwent radical hysterectomy with pelvic and paraaortic lymphadenectomy for treating early-stage and locally advanced cervical cancer were included. Among the 136 patients, 76 patients underwent preoperative ^18^F-FDG PET/CT and were enrolled in this study. The enrolled patients were semirandomly divided into a training dataset (51 patients) and a test set (25 patients) using the “doBy” R package while preserving the distribution of ITB status. Patients with a history of preoperative chemotherapy, radiotherapy, or synchronous malignancies were excluded. The patients were clinically staged according to the 2009 International FIGO staging system [18].

### 2.2. Histopathological Evaluation

Specimens were examined from multiple sections of the whole tumor areas and stained with H&E. For each case, all available specimens were independently reviewed for the detailed histopathological features and the quantitative assessment of TB by two pathologists (J.Y.P and J.Y.P) in a blinded manner, with no knowledge of the clinicopathological data and outcomes.

The pathological parameters included tumor size, FIGO stage, histological subtype, deep stromal invasion, LVI, parametrial invasion, lymph node metastasis, and the number and distribution of TB. TB was defined as an isolated single cancer cell or small cell clusters composed of ≤4 tumor cells located at the advancing edge (PTB) and within the tumor area (ITB).

### 2.3. ^18^F-FDG PET/CT Image Acquisition

All patients fasted for at least 6 h, and their blood glucose levels were determined before the administration of ^18^F-FDG. Patients with blood glucose levels >150 mg/dL were rescheduled for a later examination, and treatment was administered to maintain a blood glucose concentration <150 mg/dL in all participants. Patients received intravenous injections of approximately 5.2 MBq of FDG per kg of body weight and were advised to rest for 1 h before undergoing ^18^F-FDG PET/CT imaging. The ^18^F-FDG PET/CT scans were performed using a Discovery 600 (GE Healthcare, Chicago, IL, USA). Before the PET scan, for attenuation correction, a low-dose CT scan was obtained without contrast enhancement from the skull base to the thigh while the patient was in the supine position and breathing quietly. PET scans were also obtained from the skull base to the thigh at 2.5 min per bed position. PET images were reconstructed using a 128 × 128 matrix and an ordered-subset expectation maximum iterative reconstruction algorithm.

### 2.4. Image Interpretation and PET Image Analysis

The ^18^F-FDG PET/CT images were interpreted by two experienced nuclear medicine physicians (S.Y.J and S.W.L), and a final consensus was achieved for all patients. A positive finding in the uterine cervix was defined as any focus with increased FDG uptake compared to the surrounding normal tissue. Foci of FDG uptake mimicking positive findings in the pelvis, such as urinary activity or a functional ovarian cyst, were excluded from the analysis.

All image analyses were performed using the Advantage Workstation 4.5 software (GE Medical Systems, Waukesha, WI, USA). The primary tumor lesion was delineated by the volume of interest by using an isocontour threshold method based on the SUV, and metabolic PET parameters were assessed. SUVmax values were based on body weight and were calculated using the following formula: SUVmax = maximum activity in the region of interest (ROI) (MBq/g)/(injected dose (MBq)/bodyweight (g)). SUVmax was designated as the highest value of SUVmax of the primary tumor. The MTV was determined as the volume of voxels with an SUV threshold of the mediastinal blood pool because the mediastinal blood pool is regarded as the preferred site for measuring background activity [19]. The mean SUV of the mediastinal SUV values was determined by drawing an ROI over contiguous slices on the descending aorta, carefully excluding the walls from the ROI. The mean SUV of the mediastinal background plus 2 SDs was used as the threshold to automatically calculate the MTV [19]. The TLG was calculated as the MTV multiplied by SUVmean of the lesion. The MTV and TLG were also obtained for the primary tumor.

### 2.5. Statistical Analysis

The differences between subsets were evaluated with a Student’s *t*-test or Mann–Whitney test, and differences between proportions were compared with the chi-square test or Fisher’s exact test. Receiver operating characteristic (ROC) curve analysis was performed to identify an optimal cutoff value of metabolic parameters and radiomic finding of ^18^F-PET/CT for predicting the ITB status. Multiple logistic regression analysis was used to evaluate the metabolic parameters and radiomic findings of ^18^F-PET/CT for ITB status. The estimated odds ratios (ORs), with 95% confidence intervals (95% CIs), are presented. All statistical tests were two-sided, and *p* < 0.05 was considered significant. Statistical analysis was performed using SPSS software version 22.0 (SPSS, Chicago, IL, USA), Medcalc version 15.4 (Medcalc Software, Ostend, Belgium), and R version 3.6.3 (R Foundation for Statistical Computing, Vienna, Austria). The R packages “caret”, “glmnet”, “MASS”, and “pROC” were used for analysis.

### 2.6. Radiomic Analysis

Radiomic features were extracted using the LIFEx package (http://www.lifexsoft.org, accessed in 1 July–30 September 2020) [20]. LIFEx was set up using the following input parameters for calculating the features: 64 Gy levels to resample the ROI content, which was performed in absolute terms between a minimum of 0 and a maximum of 20 [21]. A total of 59 features were extracted from the analysis of the volumes inspected; these indices included conventional parameters, shape and size features, histogram-based features, and second- and high-order-based features. The correction for the partial volume effect was not applied. The analysis included all primary tumor lesions, irrespective of their volume; however, LIFEx calculates the shape and size indices as well as the second-order- (gray-level co-occurrence matrix (GLCM)) and high-order-based (neighborhood gray-level different matrix (NGLDM), gray-level run-length matrix, (GLRLM) and gray-level zone-length matrix (GLZLM)) features only for an ROI of at least 64 voxels due to technical reasons. The features calculated are summarized in Table S1.

Each feature value was normalized using z-score normalization (z = [x − mean {x}/SD {x}] [standard deviation {SD}]). The feature selection process consisted of two steps in the training dataset. First, a *t*-test was performed to screen potential features throughout radiomic features and conventional metabolic parameters. Only features with *p* < 0.05 were considered significant and entered into the next selection step. Least absolute shrinkage and selection operator (Lasso) regression was used to select key features to build a prediction model, with three-fold cross-validation.

Following feature selection, the prediction models were constructed using a random forest (RF), a support vector machine (SVM), and a neural network (NN) using the training dataset. We built prediction models using only conventional metabolic parameters and both conventional metabolic parameters and radiomic features. The constructed model performance was validated independently in the test dataset by the area under the ROC curve. The improvement of prediction accuracy was assessed with the net reclassification improvement (NRI) and integrated discrimination improvement (IDI) statistics. The R packages “randomForest”, “kernlab”, “neuralnet”, and “PredictABEL” were used to build and evaluate the prediction model.

## 3. Results

### 3.1. Clinicopathologic Features and Treatment Outcomes

The clinicopathologic characteristics of the study participants are listed in Table 1. The predominant FIGO stage was IB1 (*n* = 43 (56.6%)), followed by IB2 (*n* = 14 [18.4%)), IIB (*n* = 10 (13.2%)), and IIA (*n* = 9 (11.8%)). The histologic cervical cancer types were as follows: squamous cell carcinoma (*n* = 91 (66.9%)), adenocarcinoma (*n* = 37 (27.2%)), and adenosquamous carcinoma (*n* = 8 (5.9%)) (Table 1). The median ITB count was 3.5 (range, 0–40), and the median PTB count was 4 (range, 0–44). ITB and PTB were observed in 47 (61.8%) and 62 patients (81.6%), respectively.

### 3.2. Comparison of Metabolic Parameters and Radiomic Features of ^18^F-PET/CT According to TB Status

**Figure 1:**
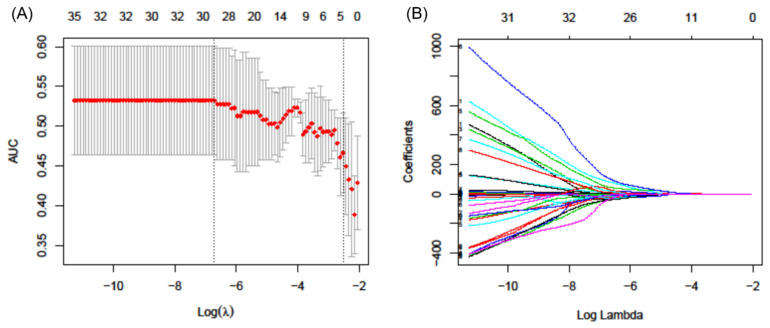
F-18 fluorodeoxyglucose positron emission tomography/computed tomography radiomic feature selection performed by the least absolute shrinkage and selection operator (Lasso) regularization method. (**A**) Area under the curve was drawn versus log (λ) by the 5-fold cross-validation. The vertical dotted line defines the optimal λ value. The optimal λ of 0.0012, with log (λ) of −6.7061 was selected; (**B**) Lasso coefficient profiles of the 48 potential PET features as selected by the *t*-test. Twenty-seven features were selected with the optimal λ.

The median SUVmax was significantly higher in the positive ITB group than in the negative ITB group (11.35 vs. 8.37, *p* = 0.0406; Figure 1). However, the median SUVmax did not change significantly according to the PTB status. Among the radiomic features, entropyGLCM (GLCM; *p* = 0.0111), coarseness (NGLDM; *p* = 0.0497), low gray-level run emphasis/long-run low gray-level emphasis (GLRLM; *p* = 0.0189 and *p* = 0.0101, respectively), low gray-level zone emphasis/short-zone low gray-level emphasis/zone-length nonuniformity zone (GLZLM; *p* = 0.0137, *p* = 0.0154, and *p* = 0.0056, respectively), sphericity/compacity (shape and size; *p* = 0.0065 and *p* = 0.0108, respectively), and kurtosis/entropy_Hist_/energy_Hist_ (histogram; *p* = 0.0267, *p* = 0.0130, and *p* = 0.0200, respectively) were significantly different according to the ITB status. However, there were no significantly different radiomic findings according to the PTB status (Table 2).

### 3.3. Multiple Logistic Regression Analysis for ITB Status

Univariate and multivariate analyses were performed to evaluate the correlation between ^18^F-FDG PET/CT values and the ITB status (Table 3). Among the significant parameters of conventional metabolic parameters (SUVmax, MTV, and TLG) and each radiomic finding (GLCM, NGLDM, GLZLM, shape and size, and histogram) in univariate analysis, the most significant parameters (the lowest *p* value) were included in multivariate analysis for the inhibition of conflicting each parameter. In univariate analysis, SUVmax (OR, 3.34; 95% CI, 1.27–8.79; *p* = 0.0146), TLG (OR, 4.42%; 95% CI, 1.33–14.72; *p* = 0.0154), entropyGLCM (OR, 5.36; 95% CI, 1.95–14.69; *p* = 0.0011), coarseness (OR, 3.94; 95% CI, 1.41–11.03; *p* = 0.0090), low gray-level run emphasis (OR, 3.45; 95% CI, 1.26–9.47; *p* = 0.0161), long-run low gray-level emphasis (OR, 3.34; 95% CI, 1.27–8.79; *p* = 0.0146), low gray-level zone emphasis (OR, 4.05; 95% CI, 1.52–10.82; *p* = 0.0052) short-zone low gray-level emphasis (OR, 3.94; 95% CI, 1.41–11.03; *p* = 0.0090), zone-length nonuniformity zone (OR, 9.16; 95% CI, 1.27–8.79; *p* = 0.0146), sphericity (OR, 6.09; 95% CI, 2.15–17.28; *p* = 0.0007), compacity (OR, 8.73; 95% CI, 2.48–30.76; *p* = 0.0007), kurtosis (OR, 3.96; 95% CI, 1.38–11.38; *p* = 0.0106), Entropy_Hist_ (OR, 5.98; 95% CI, 2.12–16.86; *p* = 0.0007), and Energy_Hist_ (OR, 5.98; 95% CI, 2.12–16.86; *p* = 0.0007) were significant parameters that correlated with positive ITB. Multivariate analysis with the entered methods showed that only compacity (OR, 5.00; 95% CI, 1.16–21.53; *p* = 0.0305) remained an independent parameter correlated with positive ITB (Table 3).

### 3.4. Predicting Model for ITB Status Using Radiomic Features of ^18^F-FDG PET/CT

**Figure 2:**
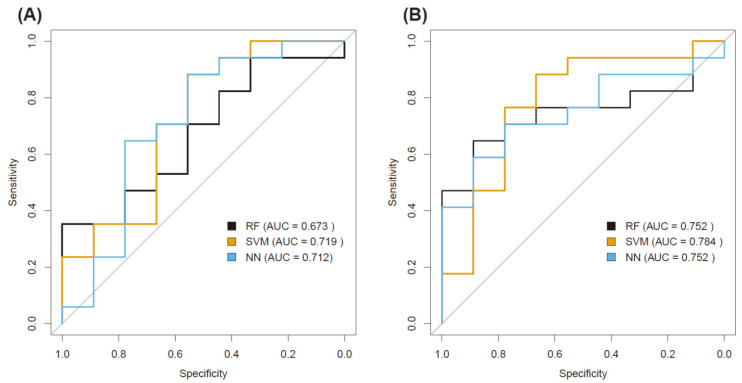
Receiver operating characteristic curves of the prediction models constructed by the random forest, support vector machine, and neural network algorithms using conventional metabolic parameters only (**A**) and conventional metabolic parameters + radiomic features (**B**) in the test dataset.

Forty-eight features were significant parameters in the *t*-test and were subjected to a further selection step by the Lasso regularization (Figure 1). Among them, the final 27 remaining features (SUVmax, MTV, SUV_Skewness, discretized_SUVmax, discretized_SUV_Skewness, discretized_SUV_Kurtosis, discretized_SUVpeak_Sphere, discretized_HISTO_ExcessKurtosis, GLCM_Entropy_log2, GLCM_Dissimilarity, GLRLM_LRE, GLRLM_LGRE, GLRLM_SRHGE, GLRLM_LRLGE, GLRLM_GLNU, GLRLM_RLNU, NGLDM_Contrast, NGLDM_Busyness, GLZLM_SZE, GLZLM_LZE0, GLZLM_LGZE, GLZLM_HGZE, GLZLM_SZLGE, GLZLM_SZHGE, and GLZLM_LZLGE) were selected. Supplementary Figure S1 shows the ROC curves of the prediction models by three machine learning algorithms in the training and test datasets. The area under the curve (AUC) values of the prediction models constructed by the RF, SVM, and NN were 0.752, 0.784, 0.752, respectively in the test dataset. Figure 2 shows the ROC curves of the prediction models built using only conventional metabolic parameters and conventional metabolic parameters+radiomics features in the test datasets. The area under the curve (AUC) values of the prediction models using conventional metabolic parameters constructed by the RF, SVM, and NN were 0.673, 0.719, and 0.712, respectively (Table 4). With the addition of radiomic features, AUC values in the test dataset were 0.752, 0.784, 0.752 in the RF, SVM, and NN models, respectively. The NRI values for the addition of radiomic features to conventional metabolic parameters were 0.183, 0.105, and 0.275 in RF, SVM, and NN models, respectively. This consisted of a respective 18.3%, 10.5%, and 27.5% improvement in classification by adding radiomic parameters into prediction models, although these gains were not statistically significant.

## 4. Discussion

In this study, the radiomic features of ^18^F-FDG PET/CT were associated with TB status, especially in the ITB status. Among the 59 features, 12 features were significantly different according to the ITB status in univariate logistic regression analysis. Among these 12 features, compacity was the most significant parameter for the ITB status in multivariate logistic regression analysis. Moreover, we developed the prediction model for the ITB status by the 3 commonly used machine learning classifiersusing conventional metabolic parameters and radiomic features, and the mean AUC was 0.763.

Radiomics is a relatively new and evolving field in medical imaging in which many features are extracted from medical image analysis and interpretation using bioinformatic approaches [15]. Furthermore, radiomics precision medicine and tumor heterogeneity have recently become a hot topic in oncological medicine [22]. At the biological level, it has been recognized that the heterogeneity of the tumor microenvironment might be reflected in medical images, with respect to cellular density, proliferation, angiogenesis, hypoxia, receptor expression, necrosis, fibrosis, and inflammation, all of which may contribute to a more aggressive phenotype and poor treatment responses [23]. Therefore, the radiomic signature may represent a segmentation of tumor subregions with different biological characteristics and contribute to treatment response and prognosis.

In colorectal cancer, high ITB has been shown to correlate with higher tumor grade, higher pT stage, lymphatic invasion, vascular invasion, nodal metastasis, and shorter survival time [24]. Moreover, ITB may lead to more aggressive clinicopathologic characteristics through a similar mechanism to that of PTB, such as increased extracellular matrix degradation, increased migration, and loss of cell adhesion [16]. Therefore, we hypothesized that ITB may be associated with higher metabolic parameters due to its aggressive nature.

To date, only one study has demonstrated a correlation between tumor cancer cell metabolism and the morphological features of aggressiveness as assessed by microscopy such as TB [24]. In an earlier study, the MTV was higher in the TB group than in the nonTB group, with marginal significance (*p* = 0.06) in laryngeal and pharyngeal carcinoma [25]. In this study, SUVmax was significantly different according to the ITB status (*p* = 0.0406). Moreover, SUVmax and TLG were associated with the ITB status in univariate logistic regression analysis (*p* = 0.0146 and *p* = 0.0154, respectively). The aggressive nature of ITB may present as higher metabolic parameters in ^18^F-FDG PET/CT.

EMT of primary tumor tissues may lead to the loss of cell-to-cell adhesion and occurrence ITB. Consequently, ITB may lead to the segmentation of tumor subregions with different biological characteristics and may contribute to tumor heterogeneity. However, to our knowledge, no study has reported on the correlation between ITB and radiomic findings. In this study, 12 features were associated with the ITB status and compacity was a powerful biomarker representing ITB status in multivariate logistic regression analysis. Previous studies demonstrated that among the radiomic features, compacity was the most significant covariate to predict local control and survival in hepatocellular carcinoma [26].

The results of our previous study showed that tumors with high TB were significantly associated with LVI, deep stromal invasion, parametrial invasion, and lymph node metastasis in cervical cancer [6]. Preoperative prediction of the TB status may help in the development of personalized medicine, such as decisions on the radicality of surgery or the extent of lymphadenectomy. However, TB status was finally determined by postoperative surgical specimens. To date, there is no modality that can estimate TB status preoperatively. Therefore, prediction models were constructed for ITB status using both conventional metabolic parameters and radiomic features from ^18^F-FDG PET/CT scans. Among the 48 features, which were significant parameters in the univariate logistic regression, 27 features were selected by the *t*-test and Lasso regularization. The AUC values of the prediction models were >0.75 in the test dataset. The prediction performance was improved in all classifiers, although they were not statistically significant. One possible reason for statistical insignificance is that the number of test dataset was too small (*n* = 25) and may underestimate the statistical power.

The main limitations of this study are its retrospective nature, heterogeneity of the sample, and small sample size, which may have contributed to selection bias. Additionally, as this is a single-center study, the generalization of our findings is limited. Despite these limitations, our study offers some unique and significant findings, in that we showed correlations between radiomic findings and the TB status for the first time and established the prediction models for the ITB status using radiomic features in ^18^F-FDG PET/CT.

In conclusion, higher metabolic quantities were observed in the positive ITB group than in the negative ITB group. Radiomic findings in ^18^F-FDG PET/CT were associated with ITB status, and among these features, compacity was the most significant covariate for the ITB status. Furthermore, prediction models for ITB status using radiomic findings in ^18^F-FDG PET/CT may contribute to personalized medicine in cervical cancer.

## Figures and Tables

**Table 1:** Clinicopathological and tumor budding characteristics.

Variables	*N* (Range)
Age (years)	
Mean ± SD	47.95 ± 10.73
Median (range)	49 (25–74)
FIGO stage (*n*, %)	
IB1	43, 56.6
IB2	14, 18.4
IIA	9, 11.9
IIB	10, 13.2
Histology (*n*, %)	
Squamous cell carcinoma	54, 71.1
Adenocarcinoma/adenosquamous carcinoma	22, 28.9
Tumor size (cm)	
Mean ± SD	3.01 ± 1.67
Median (range)	3 (0–8.5)
Lymphovascular invasion (*n*, %)	59, 77.6
Deep stromal invasion (*n*, %)	46, 60.5
Parametrial invasion (*n*, %)	28, 36.8
Lymph node metastasis (*n*, %)	22, 28.9
Tumor budding characteristics	
Intratumor budding counts	
Mean ± SD	6.40 ± 9.61
Median (range)	3.5 (0–40)
Peritumoral budding counts	
Mean ± SD	7.49 ± 9.42
Median (range)	4 (0–44)
Intratumoral budding (*n*, %)	47, 61.8
Peritumoral budding (*n*, %)	62, 81.6

FIGO = International Federation of Gynecologic Obstetrics.

**Table 2:** Comparison of radiomic features according to the tumor budding status.

	Total (*n* = 76)	Intratumor Budding			Peritumoral Budding		
Valuables		Yes (*n* = 47)	No (*n* = 29)	*p*	Yes (*n* = 62)	No (*n* = 14)	*p*
Conventional metabolic parameters							
SUVmax				0.0406			0.8828
Mean ± SD	12.30 ± 7.84	12.73 ± 6.48	11.60 ± 9.75		12.11 ± 7.69	13.11 ± 8.76	
Median (range)	10.64 (3.28–48.94)	11.35 (5.25–39.79)	8.37 (3.28–48.94)		10.50 (4.13–48.94)	13.34 (3.28–35.52)	
MTV				0.2548			0.8723
Mean ± SD	20.64 ± 29.09	23.18 ± 34.60	16.52 ± 16.45		20.78 ± 30.78	20.05 ± 20.82	
Median (range)	13.79 (2.15–220.0)	15.21 (2.44–220.00)	10.73 (2.15–76.79)		14.49 (2.15–220.00)	11.79 (6.51–76.79)	
TLG				0.0606			0.8407
Mean ± SD	170.65 ± 478.22	219.30 ± 600.25	91.81 ± 101.10		179.81 ± 526.27	130.08 ± 132.62	
Median (range)	78.75 (11.16–4118.4)	85.97 (15.76–4118.40)	57.09 (11.16–488.82)		78.74 (11.16–4118.40)	77.05 (19.25–488.82)	
Radiomic features							
GLCM				0.0111			0.8500
Entropy GLCM							
Mean ± SD	7.68 ± 1.30	7.98 ± 1.10	7.21 ± 1.46		7.70 ± 1.19	7.62 ± 1.76	
Median (range)	7.662 (4.89–10.22)	7.78 (5.85–10.22)	6.99 (4.89–9.88)		7.57 (4.98–10.22)	8.10 (4.89–9.88)	
NGLDM				0.0497			0.7276
Coarseness							
Mean ± SD	0.0204 ± 0.0157	0.0170 ± 0.0128	0.0258 ± 0.0186		0.0194 ± 0.0138	0.0249 ± 0.0225	
Median (range)	0.0140 (0.0002–0.0757)	0.0129 (0.0002–0.0514)	0.0239 (0.0025–0.0757)		0.0147 (0.0002–0.0560)	0.0125 (0.0054–0.0757)	
GLRLM				0.0189			0.0939
Low Gray-level Run Emphasis							
Mean ± SD	0.0073 ± 0.0040	0.0065 ± 0.0034	0.0087 ± 0.0044		0.0068 ± 0.0034	0.0096 ± 0.0054	
Median (range)	0.0071 (0.0013–0.0213)	0.0057 (0.0013–0.0160)	0.0081 (0.0020–20.0213)		0.0062 (0.0013–0.0160)	0.0081 (0.0020–0.0213)	
Long-Run Low Gray-level Emphasis				0.0101			0.1266
Mean ± SD	0.0098 ± 0.0064	0.0085 ± 0.0056	0.0120 ± 0.0071		0.0090 ± 0.0054	0.0134 ± 0.0093	
Median (range)	0.0087 (0.0014–0.0322)	0.0069 (0.0014–0.0299)	0.0099 (0.0022–0.0322)		0.0084 (0.0014–0.0299)	0.0101 (0.0022–0.0322)	
GLZLM				0.0137			0.2547
Low Gray-level Zone Emphasis							
Mean ± SD	0.0079 ± 0.0051	0.0069 ± 0.0045	0.0096 ± 0.0061		0.0074 ± 0.0040	0.0104 ± 0.0081	
Median (range)	0.0071 (0.0014–0.0327)	0.0060 (0.0014–0.0206)	0.0085 (0.0021–0.0327)		0.0070 (0.0014–0.0206)	0.0079 (0.0021–0.0327)	
Short-Zone Low Gray-level Emphasis				0.0154			0.2177
Mean ± SD	0.0045 ± 0.0026	0.0039 ± 0.0019	0.0054 ± 0.0033		0.0042 ± 0.0020	0.0058 ± 0.0043	
Median (range)	0.0039 (0.0008–0.0183)	0.0036 (0.0011–0.0100)	0.0047 (0.0084–0.0183)		0.0038 (0.0008–0.0100)	0.0043 (0.0016–0.0183)	
Zone Length Nonuniformity Zone				0.0056			0.2492
Mean ± SD	12.95 ± 11.89	15.47 ± 13.76	8.88 ± 6.28		13.53 ± 12.55	10.42 ± 8.22	
Median (range)	9.63 (1.88–80.85)	11.86 (3.40–80.85)	7.55 (1.88–32.24)		9.63 (1.88–80.85)	8.58 (2.77–32.24)	
Shape and Size				0.0065			0.9040
Sphericity							
Mean ± SD	5208.48 ± 4024.88	5676.9 ± 4507.4	3963.1 ± 2723.4		5238.6 ± 4203.5	5075.1 ± 3244.7	
Median (range)	4249.8 (714.7–26258.1)	4894.7 (1227.2–26258.8)	3319.1 (714.7–11649.2)		4362.1 (1122.0–26258.8)	4005.5 (714.7–11649.2)	
Compacity				0.0108			0.4859
Mean ± SD	4.44 ± 1.32	4.75 ± 1.34	3.95 ± 1.16		4.42 ± 1.34	4.52 ± 1.29	
Median (range)	4.27 (2.27–8.88)	4.58 (2.76–8.88)	3.77 (2.27–6.28)		4.23 (2.39–8.88)	4.35 (2.27–6.28)	
Histogram							0.7682
Kurtosis							
Mean ± SD	3.00 ± 0.94	2.81 ± 0.68	3.30 ± 1.21		2.94 ± 0.80	3.25 ± 1.42	
Median (range)	2.72 (1.76–7.47)	2.63 (1.76–5.05)	2.84 (2.12–7.47)		2.72 (1.76–5.96)	2.69 (2.15–7.47)	
Entropy_Hist_							0.9706
Mean ± SD	4.33 ± 0.76	4.50 ± 0.63	4.06 ± 0.88		4.33 ± 0.69	4.34 ± 1.05	
Median (range)	4.40 (2.68–5.74)	4.50 (3.18–5.74)	4.01 (2.68–5.49)		4.36 (2.68–5.74)	4.77 (2.71–5.49)	
Energy_Hist_							0.7579
Mean ± SD	0.0685 ± 0.0403	0.0591 ± 0.0320	0.0836 ± 0.0478		0.0670 ± 0.0372	0.0749 ± 0.0534	
Median (range)	0.0576 (0.0212–0.1820)	0.0518 (0.0212–0.1816)	0.0722 (0.0244–0.1820)		0.0603 (0.0212–0.1820)	0.0474 (0.0244–0.1685)	

GLCM = gray-level co-occurrence matrix; GLRLM = gray-level run-length matrix; GLZLM = gray-level zone-length matrix; MTV = metabolic tumor volume; NGLDM = neighborhood gray-level different matrix; SUVmax = maximum standardized uptake; TLG = total lesion glycolysis.

**Table 3:** Multiple logistic regression analysis for the evaluation of the correlation between radiomic features and intratumor budding status.

	Univariate Analysis			Multivariate Analysis		
Variables	Odds Ratio	95% CI	*p*	Odds Ratio	95% CI	*p*
Conventional metabolic parameters						
SUVmax (>8.858)	3.3393	1.2685–8.7908	0.0146	2.4973	0.8933–6.9810	0.0810
MTV (cm^3^, >16.3)	2.5385	0.9092–7.0869	0.0753			
TLG (>32.0247)	4.4211	1.3282–14.7159	0.0154			
Radiomic features						
GLCM						
EntropyGLCM (>7.1782)	5.3554	1.9520–14.6928	0.0011	0.6139	0.0708–5.3208	0.6579
NGLDM						
Coarseness (≤0.0254)	3.9407	1.4085–11.0251	0.0090	1.0604	0.2057–5.4660	0.9441
GLRLM						
Low Gray-level Run Emphasis (≤0.0081)	3.4533	1.2588–9.4739	0.0161			
Long-Run Low Gray-level Emphasis (≤0.0094)	3.3393	1.2685–8.7908	0.0146	1.6936	0.4822–5.9481	0.4111
GLZLM						
Low Gray-level Zone Emphasis (≤0.0074)	4.0533	1.5196–10.8155	0.0052			
Short-Zone Low Gray-level Emphasis (≤0.005)	3.9407	1.4085–11.0251	0.0090			
Zone Length Nonuniformity Zone (>14.1652)	9.1607	1.9444–43.1600	0.0051	5.3971	0.9464–30.7790	0.0577
Shape and Size						
Sphericity (>4160.0834)	6.0893	2.1463–17.2763	0.0007			
Compacity (>3.4057)	8.7344	2.4798–30.7648	0.0007	5.0047	1.1636–21.5253	0.0305
Histogram						
Kurtosis (≤3.1264)	3.9609	1.3783–11.3826	0.0106			
Entropy_Hist_ (>4.0608)	5.9815	2.1219–16.8615	0.0007			
Energy_Hist_ (≤0.0688)	5.9815	2.1219–16.8615	0.0007	2.9011	0.4526–18.5937	0.2611

CI = confidence interval GLCM = gray-level co-occurrence matrix; GLRLM = gray-level run-length matrix; GLZLM = gray-level zone-length matrix; MTV = metabolic tumor volume; NGLDM = neighborhood gray-level different matrix; SUVmax = maximum standardized uptake; TLG = total lesion glycolysis.

**Table 4:** Performance metrics of prediction models for predicting tumor budding status in the test dataset.

Model	AUC (95% CI)	NRI (95% CI)	*p*	IDI (95% CI)	*p*
Conventional metabolic parameters					
RF	0.673 (0.454–0.893)	Reference	–	Reference	–
SVM	0.719 (0.488–0.950)	Reference	–	Reference	–
NN	0.712 (0.469–0.956)	Reference	–	Reference	–
Conventional metabolic parameters + radiomic features					
RF	0.752 (0.561–0.943)	0.183 (−0.115–0.482)	0.229	0.183 (−0.129–0.495)	0.250
SVM	0.784 (0.576–0.993)	0.105 (−0.334–0.543)	0.640	0.105 (−0.359–0.543)	0.658
NN	0.752 (0.561–0.942)	0.275 (−0.158–0.707)	0.214	0.275 (−0.178–0.727)	0.234

AUC = area under the curve; CI = confidence interval; NRI = net reclassification improvement; IDI = integrated discrimination improvement; RF = random forest; SVM = support vector machine; NN = neural network.

## Data Availability

The data presented in this study are available on request from the corresponding author. The data are not publicly available due to privacy and ethical restrictions.

## Supplementary Materials

The following are available online at https://www.mdpi.com/article/10.3390/diagnostics11081517/s1, Figure S1. Receiver operating characteristic curves of the prediction models constructed by the random forest, support vector machine, and neural network algorithms using metabolic and radiomic features in the training dataset (A) and test dataset (B). Table S1: Radiomic features calculated from 18F-FDG PET/CT images in Lifex.
