# Supplementary material for: Prediction Model for Tumor Budding Status Using the Radiomic Features of F-18 Fluorodeoxyglucose Positron Emission Tomography/Computed Tomography in Cervical Cancer
Source: Diagnostics (Basel). 2021 Aug 23;11(8):1517. doi: 10.3390/diagnostics11081517 (PMC8392321; doi:10.3390/diagnostics11081517)
Supplement: Supplementary file 1 [file diagnostics-11-01517-s001.zip › diagnostics-1239510-SI.pdf]

Table S1. Radiomic features calculated from ^{18}F-FDG PET/CT images in Lifex.

	Features
Gray-level co-occurrence matrix (GLCM)	Homogeneity<br>EnergyGLCM<br>ContrastGLCM<br>CorrelationGLCM<br>EntropyGLCM<br>Dissimilarity
Neighborhood gray-level different matrix (NGLDM)	$\text{Contrast}_{\text{NGTDM}}$<br>Coarseness
Gray-level run-length matrix (GLRLM)	Short-Run Emphasis<br>Long-Run Emphasis<br>Low Gray-level Run Emphasis<br>High Gray-level Run Emphasis<br>Short-Run Low Gray-level Emphasis<br>Short-Run High Gray-level Emphasis<br>Long-Run Low Gray-level Emphasis<br>Long-Run High Gray-level Emphasis<br>Gray-Level Non-Uniformity for run<br>Run Length Non-Uniformity<br>Run Percentage
Gray-level zone-length matrix (GLZLM)	Short-Zone Emphasis<br>Long-Zone Emphasis<br>Low Gray-level Zone Emphasis<br>High Gray-level Zone Emphasis<br>Short-Zone Low Gray-level Emphasis<br>Short-Zone High Gray-level Emphasis<br>Long-Zone Low Gray-level Emphasis<br>Long-Zone High Gray-level Emphasis<br>Gray-Level Non-Uniformity for zone<br>Zone Length Non-Uniformity Zone<br>Percentage
Shape and Size	Sphericity<br>Compacity
Histogram	Skewness<br>Kurtosis<br>$\text{Entropy}_{\text{Hist}}$<br>$\text{Energy}_{\text{Hist}}$
Conventional parameters	SUV minimum<br>SUV maximum<br>SUVmean<br>SUVstandard deviation<br>SUVpeak within a sphere of 0.5 and 1 mL volume (mL)<br>Metabolic tumor volume (MTV)<br>Total lesion glycolysis (TLG)

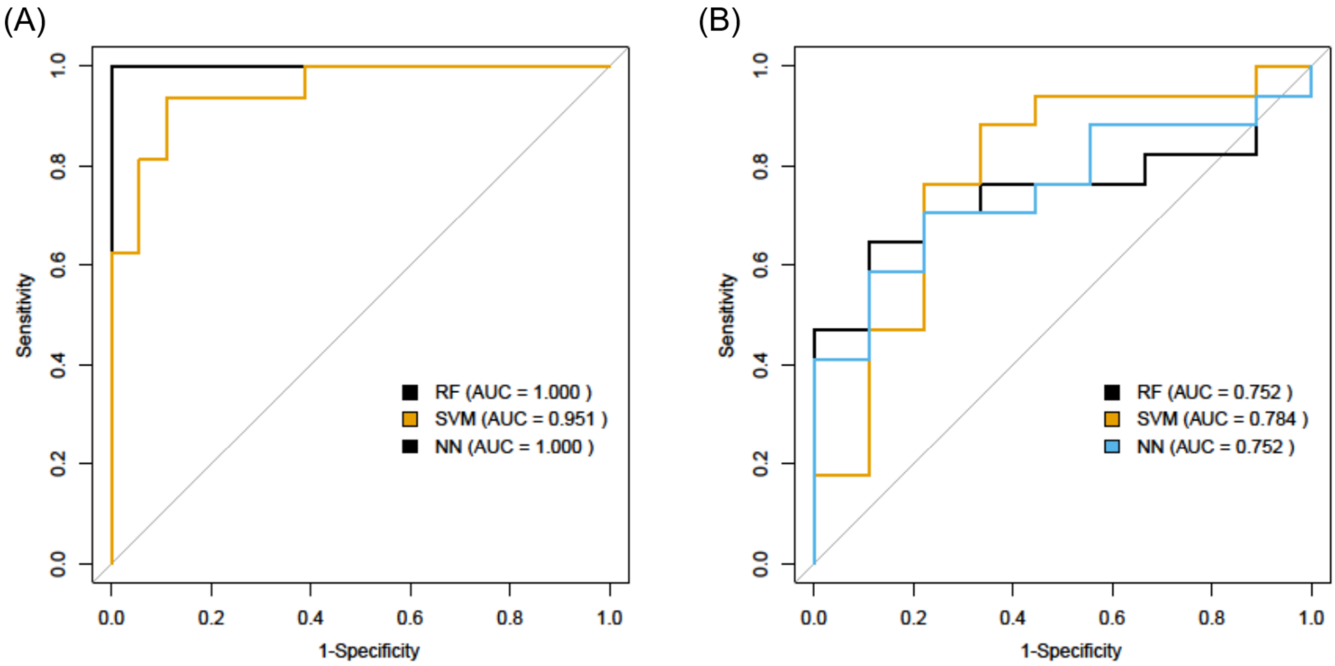

Figure S1. shows the ROC curves of the prediction models by three machine learning algorithms in the training and test datasets. The area under the curve (AUC) values of the prediction models constructed by the RF, SVM, and NN were 0.752, 0.784, 0.752 (**B**), respectively in the test dataset (**A**).
